# Global trends in research related to functional dyspepsia and anxiety or depression over the past two decades: a bibliometric analysis

**DOI:** 10.3389/fnins.2023.1218001

**Published:** 2023-11-02

**Authors:** Qian Huang, Huixiao Yuan, Qingqing Li, Yang Li, Shasha Geng, Yingqian Zhu, Min Liao, Hua Jiang

**Affiliations:** ^1^Department of General Practice, Shanghai East Hospital, Tongji University School of Medicine, Shanghai, China; ^2^Department of Geriatrics, Shanghai East Hospital, Tongji University School of Medicine, Shanghai, China

**Keywords:** functional dyspepsia, depression, anxiety, bibliometrics, research trend

## Abstract

**Background and purpose:**

Functional dyspepsia (FD) is a prevalent global disorder of the upper digestive tract characterized by functional impairment. It often coexists with anxiety/depression, significantly impairing occupational productivity and overall quality of life. This study aimed to identify emerging patterns and prominent themes within FD and anxiety/depression research through bibliometric analysis to help explore new innovative avenues for investigating this type of FD.

**Methods:**

A comprehensive review of literature encompassing FD and anxiety/depression was conducted using the Science Citation Index Extension of the Web of Science Core Collection from 2003 to 2023. Information extracted comprised “Full Record and Cited References.” Bibliometric analysis of relevant publications, including country, institution, author, journal, citations, and keywords, was conducted using CiteSpace, VOSviewer, and Bibliometrix package in R and Excel.

**Results:**

Studies related to FD and anxiety/depression have demonstrated an ascending trajectory since 2003. Our bibliometric analysis identified 338 studies published by 2023. *NEUROGASTROENTEROLOGY AND MOTILITY* emerged as the most prolific journal, while *GASTROENTEROLOGY* retained pre-eminence within the top 10 published journals. China emerged as the most prolific country, with two institutions within the top 10 in terms of volume of publications. The Mayo Clinic stood as the foremost institution in terms of publication volume, with the Chengdu University of Traditional Chinese Medicine exhibiting robust collaborative engagement. Eminent author influence was attributed to Talley NJ of Newcastle University, Australia. Clusters of extensively cited papers and prevalent keywords delineate the status and trend of FD and anxiety/depression research. This encompasses FD, anxiety, depression, sleep disorders, and functional gastrointestinal disorders. Furthermore, the timeline view map or trend-term analysis suggested that duodenal low-grade inflammation (“duodenal eosinophilia” and “mast cells”) might be a new concern associated with FD and anxiety/depression.

**Conclusion:**

Employing bibliometric analysis, this study revealed prevalent focal areas and new trends within FD and anxiety/depression research. These insights serve as valuable guidance for scholars seeking to delve into new research avenues.

## 1. Introduction

Functional dyspepsia (FD) is a prevalent functional disorder that affects the stomach and the first part of the small intestine. Common symptoms include postprandial fullness, early satiation, epigastric pain, and epigastric burning, persisting for at least 6 months to warrant a diagnosis of FD (Stanghellini et al., 2016). Based on pathophysiology and etiology, FD comprises three subtypes: postprandial distress syndrome (PDS), epigastric pain syndrome (EPS), and a subtype with overlapping PDS and EPS features (Enck et al., 2017). Being an incurable, chronic gastrointestinal dysfunction (Ford et al., 2020), FD affects work efficiency and quality of life (Aro et al., 2011; Sander et al., 2011), incurring substantial direct and indirect costs. The annual economic burden of FD in the United States (US) is estimated to reach $18.4 billion (Lacy et al., 2013). FD frequently accompanies various complications (Ford et al., 2010; Quigley and Lacy, 2013; Carbone et al., 2020), with associated comorbidities being a major determinant of escalated healthcare usage and expenses (Brook et al., 2012).

Psychological comorbidity, facilitated by intricate gut-brain communication via the enteric nervous system and hypothalamic-pituitary-adrenal axis, significantly contributes to FD development (Ford et al., 2020). Anxiety and depression hold particular relevance, exerting a significant effect on FD (Henningsen et al., 2003; Van Oudenhove and Aziz, 2013). Evidence indicates elevated levels of depression and anxiety in patients with FD compared to those with peptic ulcers and healthy volunteers (Filipovic et al., 2013). A 10-year Swedish study identified a 7.6-fold increased FD risk among individuals exhibiting anxiety at the beginning of the study (Aro et al., 2015). Such associations might be attributed to altered discomfort and pain thresholds, coupled with reduced proximal stomach compliance in anxious states (Van Oudenhove et al., 2007). Notably, amelioration of FD symptoms over 3 to 6 months correlated with reduced anxiety levels (Singh et al., 2022). Another prospective study reported that heightened depression levels at baseline were predictive of subsequent FD development over 12 years (Koloski et al., 2012). Additionally, tricyclic antidepressants as enterocranial neuromodulators stand as effective second-line FD treatments (Black et al., 2022). Overall, the relationship between FD and anxiety/depression has gained increasing attention. However, no single study has objectively summarized or analyzed research trends in this domain.

Bibliometrics is a methodology that employs mathematics and statistical principles for extensive analysis and exploration of specific topics, facilitating an in-depth understanding of research frontiers and trends (Xu H. Q. et al., 2022). This technique also aids in tracking research growth, comparing contributions across countries, institutions, authors, and journals in a given field (Gonzalez-Alcaide et al., 2016; Wu et al., 2021). CiteSpace and VOSviewer are popular software tools used for visualizing data in bibliometric analysis. CiteSpace, conceived by Chen (2004), creates visual knowledge maps revealing research trajectories, stages, and frontier characteristics of institutions and authors, offering predictive insights into a field’s developmental trends (Zhu J. et al., 2021). Meanwhile, VOSviewer, developed by the Scientific and Technological Research Centre of Leiden University, enables the construction of a visual author map or keyword map based on co-reference data or co-occurrence data (van Eck and Waltman, 2010), particularly valuable for interpreting large maps. In addition, Microsoft Excel and an online analysis platform using the “bibliometrix” package are used for bibliometric analysis (Wan et al., 2022; Zhao et al., 2022).

Therefore, this study mainly uses the above software to conduct a comprehensive and scientifically reliable bibliometric analysis of global publications associated with FD and anxiety/depression over the past two decades to promote understanding, application, and further research in related fields.

## 2. Methods

### 2.1. Sources of data and search strategies utilized

**Figure 1:**
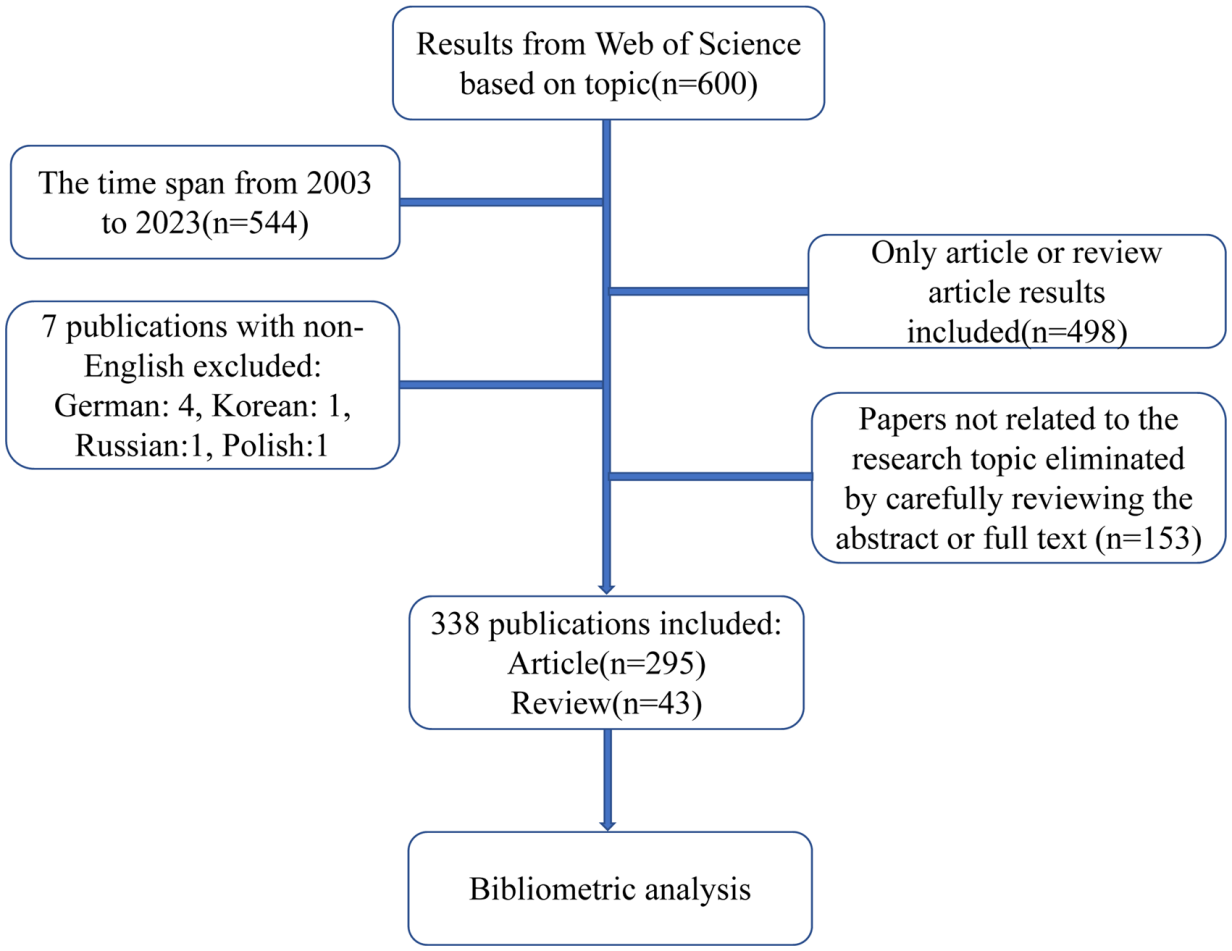
The implementation process of the study.

The study conducted an extensive search for publications associated with FD and anxiety/depression spanning 2003–2023 (up to 25 July). The Web of Science Core Collection’s (WOSCC) Science Citation Index Expanded was used to ensure publication authority and influence (Cheng et al., 2022). Employing an advanced search strategy, the following parameters were employed: Topics = (“depression” OR” depressive disorder*” OR “depressive symptom*” OR” emotional depression” OR “anxiety” OR “anxiety disorder*” OR “anxiety symptom*” OR “common mental disorder*” OR “psychiatric disorder*”) AND (“functional dyspepsia” OR “non-ulcer dyspepsia” OR “idiopathic dyspepsia”). The inclusion criteria of this study were as follows: (1) studies published between 2003 and 2023; (2) studies that were an “article” or a “review article”; (3) studies published in English only. The publications were retrieved independently and extracted by two researchers on 25 July 2023. Stringent evaluation and screening of abstracts or full texts were conducted to ensure alignment with the research topic during initial screening. The study’s workflow is illustrated in Figure 1.

### 2.2. Bibliometric analysis

For our bibliometric analysis, Microsoft Excel 2019, CiteSpace (6.2.R4), VOSviewer (1.6.18), and Bibliometrix online analysis platform were sequentially employed (Yang et al., 2022). Microsoft Excel facilitated the creation of charts depicting the annual publication volume and the annual cumulative publication volume. CiteSpace enabled the visualization of cooperative networks across countries, institutions, and journals, as well as dual-map overlays for journals. Furthermore, CiteSpace was used for cluster analysis of co-cited references, timeline view, and display of references with the strongest citation bursts. Meanwhile, VOSviewer generated visuals for author co-authorship networks, author co-citation networks, and co-occurrence density of author keywords. Bibliometrix facilitated analysis of keyword dynamics and trending topics. In these visualization maps, different nodes correspond to different countries, institutions, journals, authors, cited references, and keywords. Node size represents the frequency. The number of lines in the visual representation indicates the degree of collaboration among countries, authors, or institutions, with line thickness denoting the strength of connections. Cluster analysis incorporated evaluation metrics such as modularity Q and mean silhouette. A *Q*-value of >0.3 indicates a significant clustering structure, while a mean silhouette value of >0.5 denotes credible clustering outcomes (Liu et al., 2021).

## 3. Results

### 3.1. Publication trend analysis

**Figure 2:**
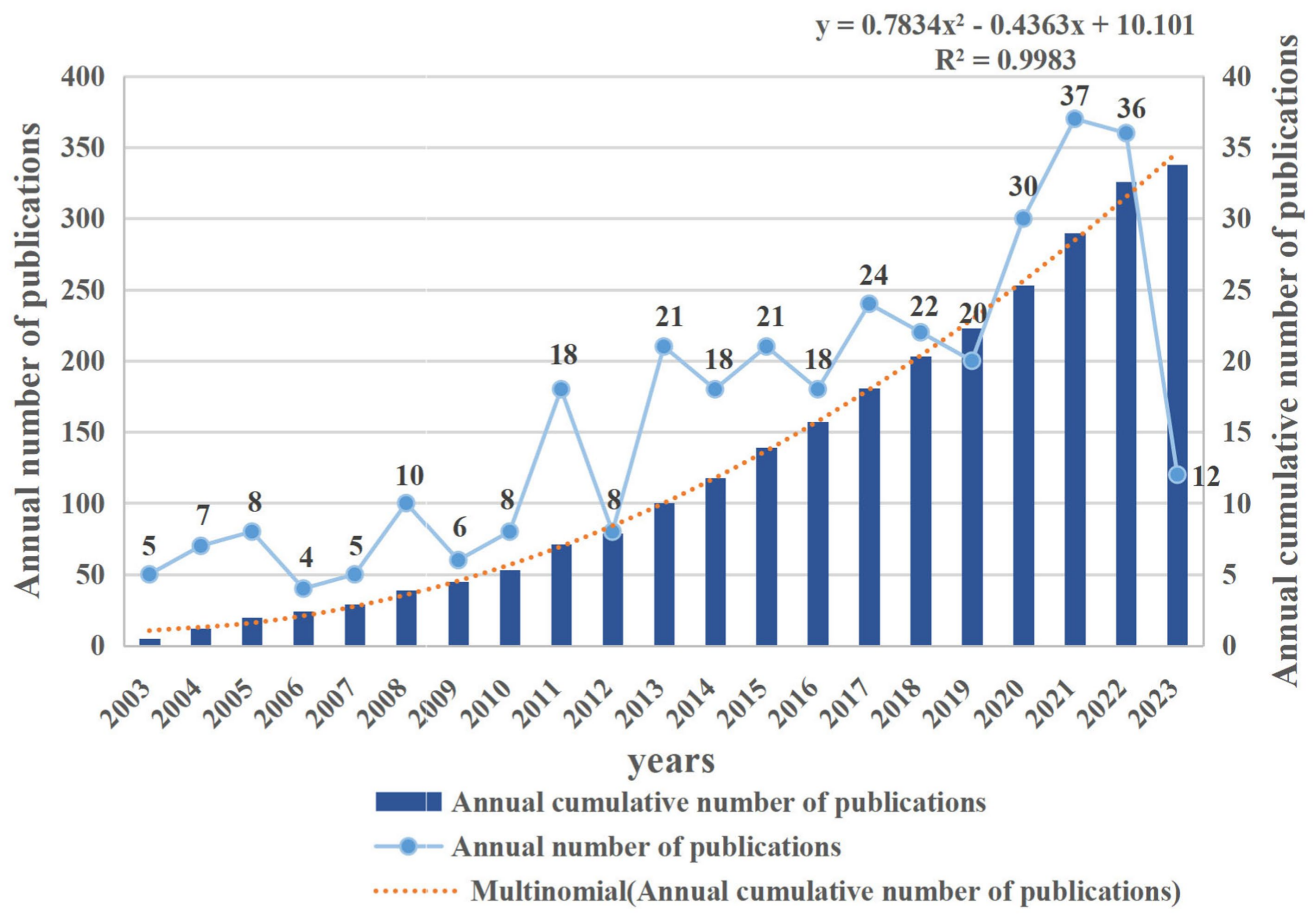
Annual and cumulative growth trend of publications associated with functional dyspepsia and anxiety/depression.

The final selection comprised 338 papers, encompassing 295 articles and 43 review articles. The progressive increase in annual publications from 2003 is depicted in Figure 2, illustrating a fluctuating upward trajectory. Notably, the year 2006 witnessed the lowest output, with only four papers addressing FD and anxiety/depression. In contrast, 2021 marked the zenith with 37 annual, nearly nine times the 2006 figure. The annual cumulative publications were also analyzed to understand the law of paper publication. This analysis revealed a growth trend accurately expressed by the exponential function *y* = 0.7834*x*^2^–0.4363*x* + 10.101(*R*^2^ = 0.9983). In conclusion, these findings underscore growing attention toward the relationship between FD and anxiety/depression.

### 3.2. Leading country analysis

**Figure 3:**
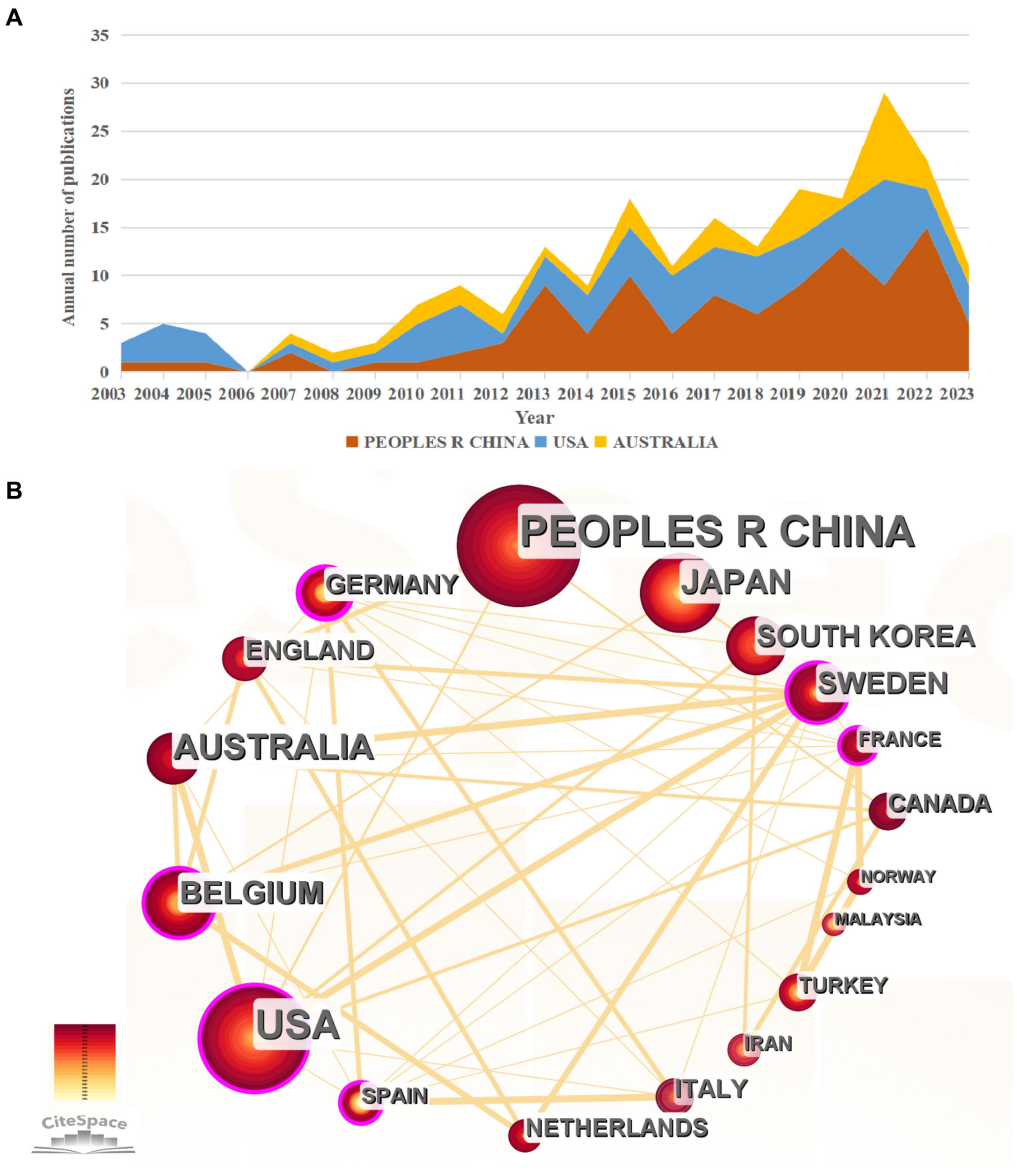
Contribution of different countries to functional dyspepsia- and anxiety/depression-related research. **(A)** Annual trends in the number of publications of the top three countries. **(B)** Cooperation chart of the top 18 countries with the number of publications (number of publications ≥5).

Publications from Taiwan were grouped under China. Over the span of 2003–2023, contributions from 42 were observed in the selected publications. Table 1 presents the top 10 countries contributing to research on FD and anxiety/depression over the last two decades. Notably, China led with 104 publications, accounting for 30.77% of the total publications, accumulating 1,520 citations at an average of 14.62 citations per paper and an H-index of 24. The US followed with 79 publications (23.37%), receiving 2,376 citations with an average of 30.08 citations per paper and an H-index of 27. Australia, ranked third with 39 (11.54%) published papers, garnering 1,757 citations at an average of 45.05 citations per paper and an H-index of 17. While China’s publication count is substantial, it does not surpass the US and Australia in terms of total citations and citations per paper, indicating a greater influence of the latter two nations in this research domain. Germany, with a modest 16 publications, achieved an impressive average of 60.69 citations per paper, potentially motivating China to publish more high-quality papers. Figure 3A illustrates the annual output trends of the three most prolific countries from 2003 to 2023. Furthermore, CiteSpace software was used to analyze and visualize international collaborations. Node sizes denote publication quantity, while the number of lines represents cooperation intensity. Among the top 18 publishing countries, robust cooperation was observed (Figure 3B). Notable cooperative partnerships emerged among Asian (e.g., China and Japan), North American (e.g., US), Oceanian (e.g., Australia), and European countries (e.g., Belgium and Sweden) constituting the cornerstone of the international collaboration landscape.

**Table 1:** Top 10 countries contributing the most to publications.

Ranking	Countries	Publications	Citation	Average citation	H-index	Year
1	PEOPLE’S REPUBLIC OF CHINA	104	1,520	14.62	24	2003
2	UNITED STATES	79	2,376	30.08	27	2003
3	AUSTRALIA	39	1,757	45.05	17	2007
4	JAPAN	34	853	25.09	15	2005
5	BELGIUM	28	1,097	39.18	17	2007
6	SOUTH KOREA	23	458	19.91	13	2010
7	SWEDEN	22	975	44.32	13	2006
8	GERMANY	16	971	60.69	12	2004
9	ENGLAND	15	348	23.20	9	2005
10	ITALY	13	314	24.15	7	2008

### 3.3. Analysis of major institutions

**Figure 4:**
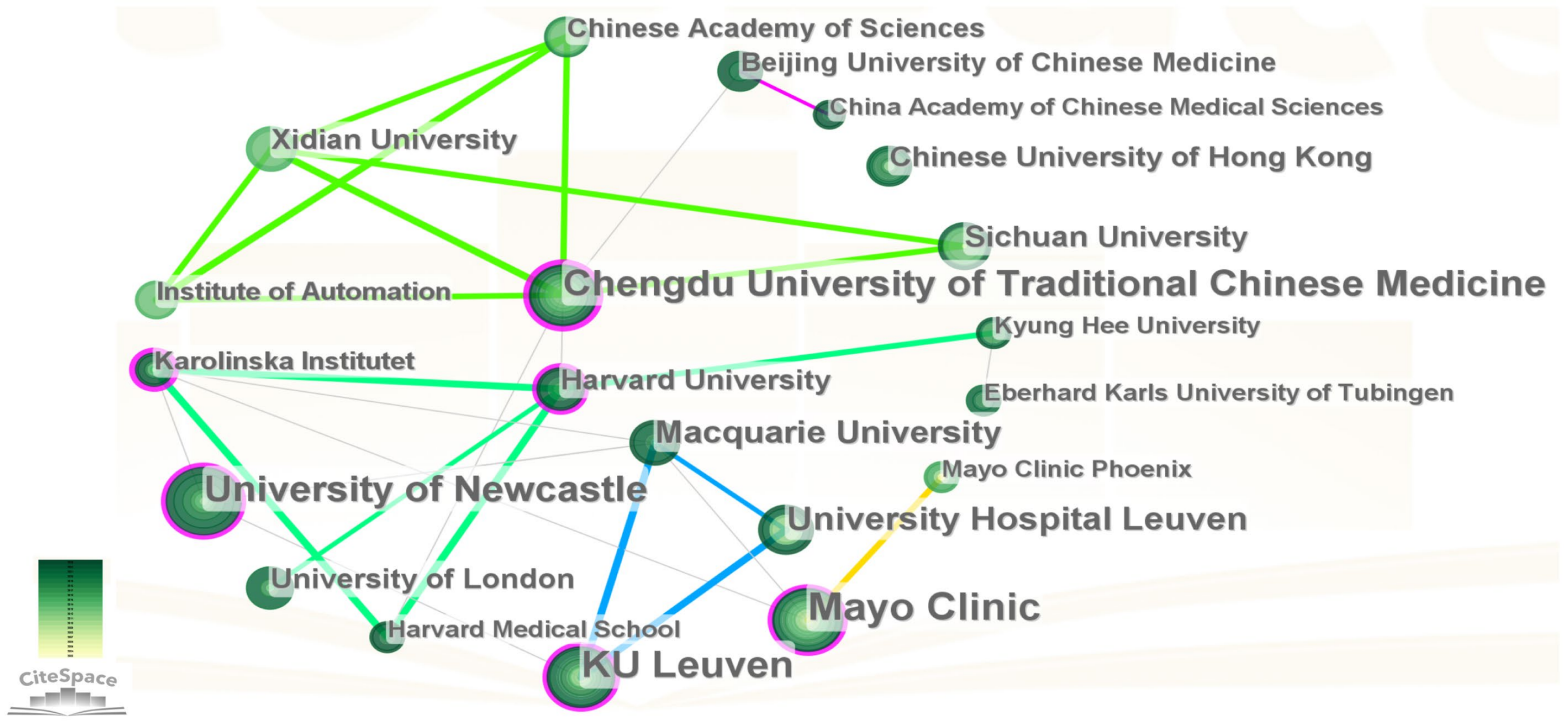
Contribution of different institutions to functional dyspepsia- and anxiety/depression-related studies (*n* = 20, number of publications >5).

Over the past two decades, 609 institutions contributed to the 338 studies concerning FD and anxiety/depression. Table 2 presents the top 10 institutions based on the publication count. The Mayo Clinic (*n* = 26), KU Leuven (*n* = 23), University of Newcastle (*n* = 21), and Chengdu University of Traditional Chinese Medicine (*n* = 20) each published over 20 papers. Intermediary centrality, an important indicator to evaluate partnership, was used. The greater the centrality, the stronger the partnership (Zhang et al., 2022). Chengdu University of Traditional Chinese Medicine, Mayo Clinic, and the University of Newcastle emerged as the leading institutions with intermediate centrality >0.1, indicating their pivotal roles as active collaborative centers. Subsequently, the collaboration networks for organizations with more than five publications were mapped using CiteSpace software. As presented in Figure 4, 20 institutions primarily formed an extensive cooperative relationship with Chengdu University of Traditional Chinese Medicine-Xidian University, University of Newcastle, Mayo Clinic, Karolinska Institute, and Harvard University, forming the core. Noteworthy partnerships include Chengdu University of Traditional Chinese Medicine-Xidian University’s close cooperation with Sichuan University, the Chinese Academy of Sciences, and the Institute of Automation. The University of Newcastle and the Mayo Clinic maintained partnerships with Karolinska Institute and Macquarie University. In addition, Macquarie University formed a close partnership with KU Leuven and University Hospital Leuven. Harvard University works closely with Karolinska Institute, University of London, Harvard Medical School, and Kyung Hee University.

**Table 2:** Top 10 institutions contributing the most to publications.

Ranking	Institutions	Publications	Centrality	Year
1	Mayo Clinic	26	0.20	2004
2	KU Leuven	23	0.11	2007
3	University of Newcastle	21	0.17	2011
4	Chengdu University of Traditional Chinese Medicine	20	0.21	2011
5	University Hospital Leuven	15	0.00	2007
6	Macquarie University	13	0.08	2012
7	Nippon Medical School	12	0.00	2009
8	Sichuan University	10	0.07	2007
9	Harvard University	9	0.15	2016
10	University of London	9	0.02	2012

### 3.4. Active author analysis

**Figure 5:**
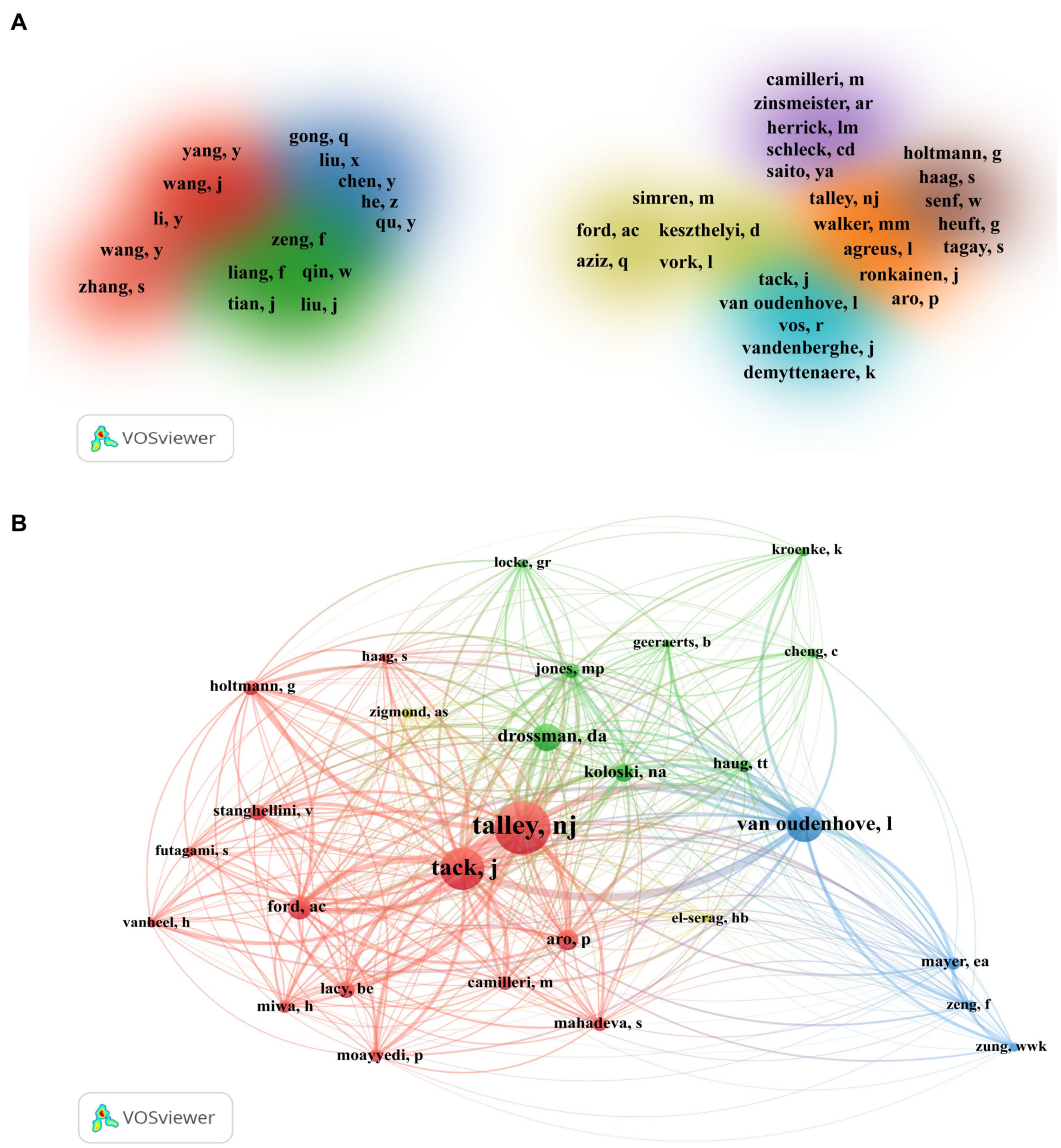
Authors involved in studies associated with functional dyspepsia and anxiety/depression. **(A)** Authors co-authorship network. The entry criteria set by VOSviewer software was ≥3 collaborations. A total of 112 authors were selected and eight clusters were obtained. One color represents a cluster, and closely related authors are assigned to the same cluster. Due to limited space, the top five authors who collaborated with other authors the most were identified. **(B)** Author co-citation analysis network. The VOSviewer software set the number of citations ≥45 as the inclusion criteria. Twenty-eight authors were selected. A node represents the author, the line between nodes represents the co-citation relationship, and the larger the node and label, the more the citation times.

Table 3 outlines the top 10 authors based on the number of publications on FD and anxiety/depression. Talley NJ leads with a substantial number of publications (*n* = 34), total citations (1,882), and an H-index of 17, significantly surpassing other authors. Zeng F and Tack J share the second position with 19 papers each. However, Tack J had a higher number of total citations (902 vs. 473) and average citations per article (47.47 vs. 24.89) than Zeng F. Remarkably, Van Oudenhove L’s 15 publications achieved an average citation count of 64.07. The co-authorship cluster density map is presented in Figure 5A using VOSviewer software. This map encompasses 112 authors with more than three publications, organized into eight distinct color-coded clusters, each displaying the five authors with the most frequent collaborations. Moreover, co-citation relationships among authors were visualized, emphasizing 28 authors cited over 45 times as pivotal researchers (Figure 5B). The circle size corresponds to the number of citations received by an author’s paper. The lines connecting the circles indicate the co-citation relationship between two authors. Total link strength (TLS) represents an author’s influence on other authors participating in the study (Zhao et al., 2022). The findings illustrated in Figure 5B highlight that Talley NJ (TLS = 5,274), Tack J (TLS = 5,047) and Van Oudenhove L (TLS = 3,717) have substantial influence over other participating authors.

**Table 3:** Top 10 authors who contributed the most publications.

Ranking	Author	Publications	Citation	Average citation	H-index	Year
1	Talley NJ	34	1882	55.35	17	2004
2	Zeng F	19	473	24.89	14	2011
3	Tack J	19	902	47.47	14	2007
4	Futagami S	16	363	22.69	10	2009
5	Van Oudenhove L	15	961	64.07	14	2007
6	Liang FR	13	387	29.77	12	2011
7	Qin W	13	378	29.08	12	2011
8	Holtmann G	12	531	44.25	9	2004
9	Kodaka Y	12	155	12.92	9	2011
10	Yamawaki H	12	135	11.25	8	2013

### 3.5. Noteworthy journals

**Figure 6:**
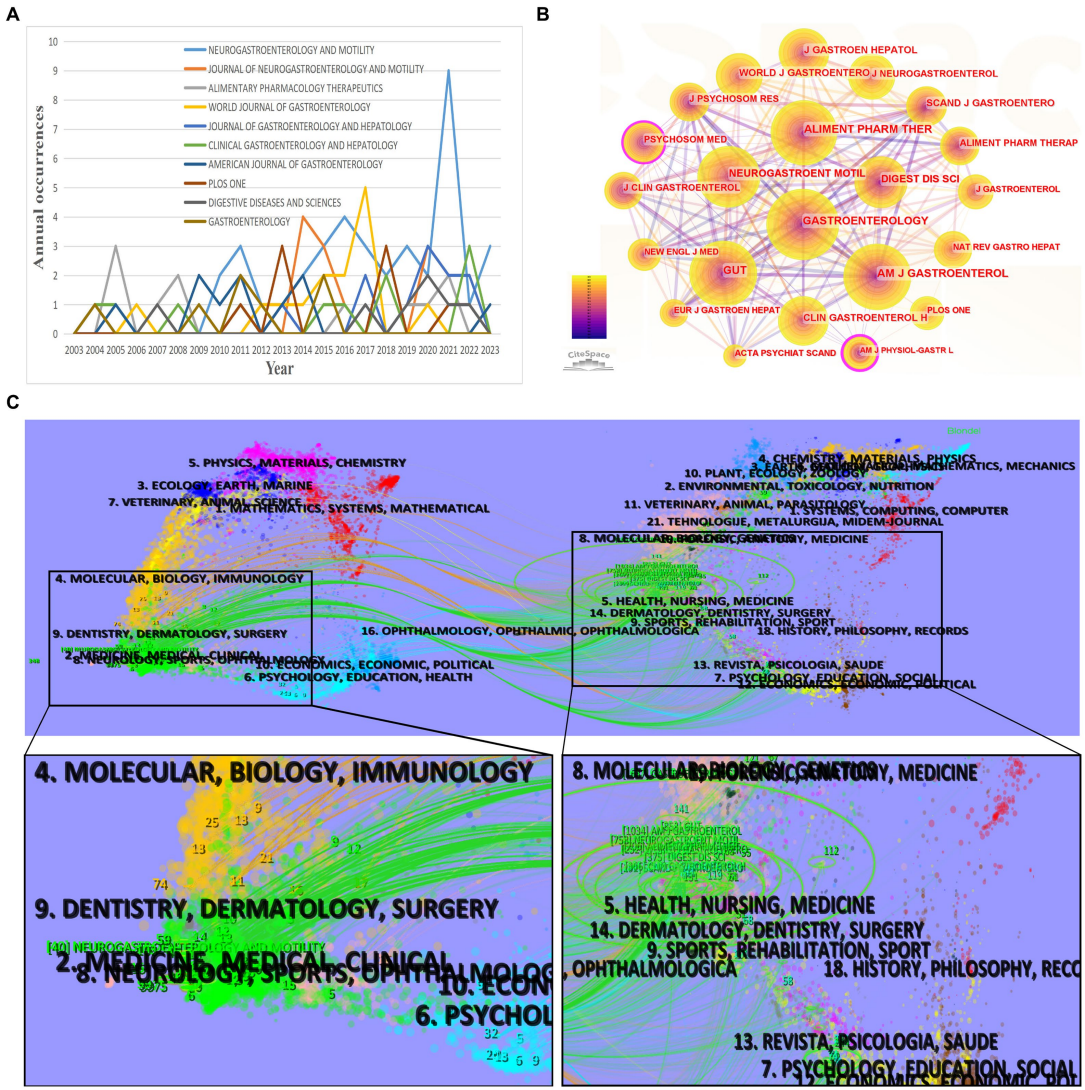
Analysis of published and cited journals of functional dyspepsia (FD)- and anxiety/depression-related studies. **(A)** Annual publication trends of the top 10 journals. **(B)** Visualization of the co-citation analysis network of 22 journals with citation times ≥60 using CiteSpace software. One node represents a journal, and the area represents the citation frequency. The size of the node and label was positively correlated with the number of citations. **(C)** A dual-map overlay drawn by CiteSpace software. On the left of the line are the published journals of FD- and anxiety/depression-related studies, and on the right of the line are the cited journals. Different colored lines indicate that the journal has a citation relationship. The disciplines of journals have been enlarged on either side of the bold lines to present the most important journal disciplines.

Among the 121 academic journals that published research articles on FD and anxiety/depression, the top 10 most prolific journals are presented in Table 4. *NEUROGASTROENTEROLOGY AND MOTILITY* ranked first in terms of publication volume (n = 40; citation times: 747; impact factor [IF] 2023 = 3.5). Notably, two of the top 10 journals, namely *GASTROENTEROLOGY* (IF 2023 = 29.4) and *CLINICAL GASTROENTEROLOGY AND HEPATOLOGY* (IF 2023 = 12.6), both based in the US, had IFs exceeding 10. While *GASTROENTEROLOGY* published only one-fifth of *NEUROGASTROENTEROLOGY AND MOTILITY’s* volume, its analysis revealed a substantial citation rate of 136.43 per article. In addition, the annual publication volume of the top 10 journals was analyzed to better understand their annual publication trends (Figure 6A). This analysis revealed that before 2016, all 10 journals maintained an annual publication volume below four. Notably, *WORLD JOURNAL OF GASTROENTEROLOGY* surpassed the threshold of four articles in 2017, while *NEUROGASTROENTEROLOGY AND MOTILITY* achieved a peak of nine articles in a single year (2021). A co-citation analysis of the 22 journals with over 60 citations was executed, visualized via CiteSpace. As presented in Figure 6B, the top three journals cited were *GASTROENTEROLOGY*, *AMERICAN JOURNAL OF GASTROENTEROLOGY*, and *GUT*.

**Table 4:** Top 10 journals with the most publications regarding anxiety/depression in functional dyspepsia.

Ranking	Journals	Publications	Citations	IF (2023)	JCR (2023)	Country
1	NEUROGASTROENTEROLOGY AND MOTILITY	40	747	3.5	Q3	England
2	JOURNAL OF NEUROGASTROENTEROLOGY AND MOTILITY	18	415	3.4	Q2	South Korea
3	ALIMENTARY PHARMACOLOGY THERAPEUTICS	14	690	7.6	Q1	England
4	WORLD JOURNAL OF GASTROENTEROLOGY	14	291	4.3	Q2	China
5	JOURNAL OF GASTROENTEROLOGY AND HEPATOLOGY	13	245	4.1	Q2	Australia
6	CLINICAL GASTROENTEROLOGY AND HEPATOLOGY	11	400	12.6	Q1	USA
7	AMERICAN JOURNAL OF GASTROENTEROLOGY	11	535	9.8	Q1	USA
8	PLOS ONE	9	160	3.7	Q2	USA
9	DIGESTIVE DISEASES AND SCIENCES	8	85	3.1	Q3	USA
10	GASTROENTEROLOGY	7	955	29.4	Q1	USA

IF, influence factor; JCR, journal citation reports; USA, United States of America.

### 3.6. A dual-map overlay of journals associated with FD and anxiety/depression

The journal’s dual map overlay reveals the overall scientific contribution. In this representation, each dot on the map represents a journal, with the network of citing journals on the left and the network of cited journals on the right. Disciplinary affiliations are indicated by labels. Connecting lines between the left and right sides of the map indicate the citation relationship between the journals, with thicker lines indicating robust knowledge flow within the cluster (Chen S. et al., 2022). As illustrated in Figure 6C, citing papers concerning FD and anxiety/depression predominantly emerge from journals affiliated with medicine/medical/clinical fields. Simultaneously, cited papers predominantly stem from journals pertaining to health/nursing/medicine and psychology/education/social disciplines.

### 3.7. Analysis of co-cited references

Highly cited references play a crucial role in disseminating fundamental knowledge, outlining research directions, and spotlighting field hotspots (Wang et al., 2021). Table 5 summarizes the top 10 extensively cited references in the context of FD and anxiety/depression. “Gastroduodenal Disorders” a work by Stanghellini et al. published in *GASTROENTEROLOGY* in 2016, claims the most frequent citations (Stanghellini et al., 2016). This literature provides several important suggestions for improving the specificity of the definition of FD subtypes. The key propositions include the following: (1) A comprehensive scope of meal-induced symptoms encompassed postprandial fullness, early satiation, epigastric pain, and epigastric burning. Therefore, PDS is characterized by dyspepsia caused by meals, while EPS does not occur only after meals. (2) The frequency of each symptom (≥3 times/week for PDS and ≥1 time/week for EPS) and minimum thresholds for severity (severe enough to interfere with daily living) are more precisely defined. The reference also introduces psychotropic drugs, particularly antidepressants, as second-line FD treatment (possibly confined to specific patient subtypes) and introduces the concept of intestinal inflammation. Significantly, this work not only contributed valuable suggestions to the establishment of the definition of FD by the Rome IV Consensus but also charted novel avenues for subsequent FD and anxiety/depression studies (Herrick et al., 2018; Ronkainen et al., 2021; Huang Q. et al., 2022). This multi-faceted impact elucidates its prominence within the field.

**Table 5:** Top 10 cited references on functional dyspepsia and anxiety/depression.

Ranking	Title	Citation	Journals	First author	Year
1	Gastroduodenal disorders	33	Gastroenterology	Stanghellini V	2016
2	Epidemiology, clinical characteristics, and associations for symptom-based Rome IV functional dyspepsia in adults in the USA, Canada, and the UK: a cross-sectional population-based study	27	Lancet Gastroenterology and Hepatology	Aziz I	2018
3	Worldwide prevalence and burden of functional gastrointestinal disorders, results of Rome Foundation global study	24	Gastroenterology	Sperber AD	2021
4	Anxiety is associated with uninvestigated and functional dyspepsia (Rome III criteria) in a Swedish population-based study	22	Gastroenterology	Aro P	2009
5	Determinants of symptoms in functional dyspepsia: gastric sensorimotor function, psychosocial factors or somatization?	20	Gut	Van Oudenhove L	2008
6	Functional dyspepsia	20	New England Journal of Medicine	Talley NJ	2015
7	Functional dyspepsia	19	Nature Reviews Disease Primers	Enck P	2017
8	Functional gastroduodenal disorders	19	Gastroenterology	Tack J	2006
9	The role of psychosocial factors and psychiatric disorders in functional dyspepsia	16	Nature Reviews Gastroenterology and Hepatology	Van Oudenhove L	2013
10	Abnormal regional brain activity during rest and (anticipated) gastric distension in functional dyspepsia and the role of anxiety: an H_2_^15^O-PET study	15	American Journal of Gastroenterology	Van Oudenhove L	2010

**Figure 7:**
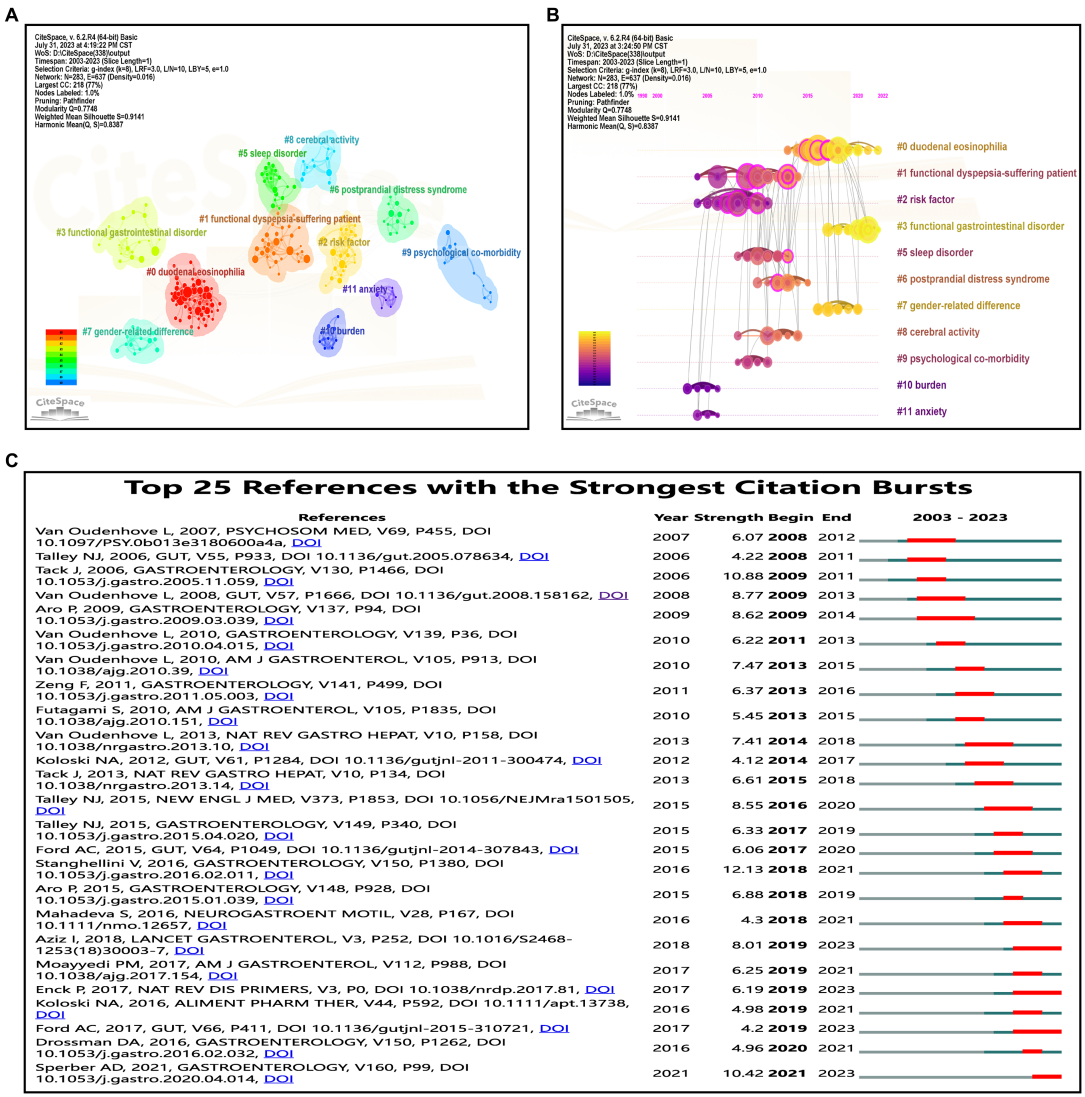
Bibliometric analysis of co-cited references associated with functional dyspepsia and anxiety/depression publications. **(A)** CiteSpace software divided the co-cited literature into 11 clusters. Different clusters are presented in different colors. The co-cited literature of the same cluster has a high degree of homogeneity. **(B)** A timeline view of 11 clusters drawn by CiteSpace software. **(C)** Analysis of the top 25 references to the strongest outbreaks using CiteSpace software. In the figure, the higher the value of strength, the stronger the influence.

Using CiteSpace software, 11 clusters for co-cited references associated with FD and anxiety/depression were generated, revealing distinct focal points and thematic bases within the field (Gonzalez-Alcaide et al., 2016). The network exhibited a modularity *Q* value of 0.7748 and an average silhouette score of 0.9141, demonstrating high reliability in clustering (Du et al., 2022). The cluster with the most cited reference was “#0 duodenal eosinophilia,” followed by “#1 functional dyspepsia-suffering patient” (Figure 7A). A timeline view was generated to understand the distribution of these clusters at different points in time (Figure 7B). Early references emphasized social psychological factors such as “burden” and “anxiety.” Subsequent literature delved into psychological comorbidity, cerebral activity, sleep disturbances, and sex-related differences associated with FD. Recent years have witnessed a surge in references primarily focused on duodenal eosinophilia and functional gastrointestinal disorders. This burst of references underscores evolving themes over time, reflecting the scientific community’s prevailing interests at various junctures (Tan et al., 2023). The top 25 references associated with the most powerful outbreaks have been compiled. As presented in Figure 7C, the strongest reference for citation outbreaks since 2008 comes from Stanghellini V’s 2016 paper entitled “Gastroduodenal Disorders” (Stanghellini et al., 2016). Securing the second spot is Tack J’s paper published in 2006 (Tack et al., 2006). Both references were instrumental in shaping the classification and definition of functional gastrointestinal disorders, including FD. Furthermore, global prevalence and the burden of functional gastrointestinal disorders have gathered significant attention from researchers.

### 3.8. Keyword analysis

**Figure 8:**
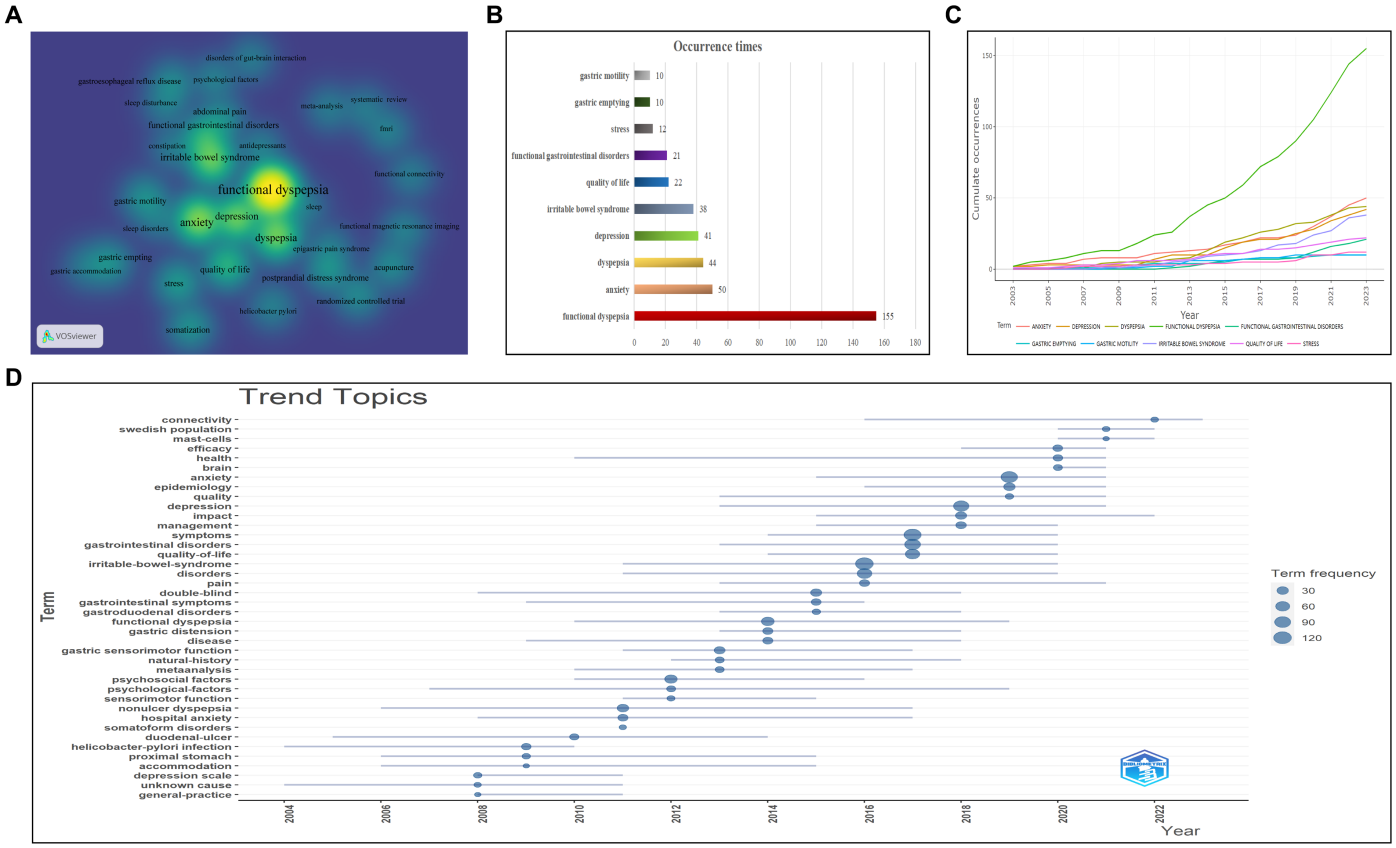
Co-occurrence analysis of keywords associated with functional dyspepsia and anxiety/depression. **(A)** Author keyword co-occurrence density map drawn by VOSviewer (*n* = 31, frequency of ≥5 times). The brighter the color, the more the co-occurrence times. **(B)** The top 10 author keywords with the highest frequency. **(C)** An online analysis platform based on the “Bibliometrix” package to analyze the cumulative annual occurrence of the top 10 author keywords. **(D)** A trend topic analysis of keywords plus through the online analysis platform.

Conducting keyword co-occurrence analysis effectively reveals prevailing hot topics within the field. A visual project density map was constructed by extracting 31 author keywords from 338 publications, each appearing at least five times (Figure 8A). The brightness of the color denotes keyword frequency, with brighter colors indicating a higher frequency and darker colors indicating a lower frequency. Notably “functional dyspepsia” was the most prevalent author keyword with significant influence on other keywords (occurrence: 155; TLS: 222). Figure 8B depicts the top 10 author keywords with the highest co-occurrence frequencies: “functional dyspepsia (*n* = 155),” “anxiety (*n* = 50),” “dyspepsia (*n* = 44),” “depression (*n* = 41),” “irritable bowel syndrome (*n* = 38),” “quality of life (*n* = 22),” “functional gastrointestinal disorders (*n* = 21),” “stress (*n* = 12),” “gastric emptying (*n* = 10),” and “gastric motility (*n* = 10).”

Frequency-over-time and trend-topics analyses through Bibliometrix’s online platform were conducted to examine the emerging trends. Figure 8C portrays a gradual pre-2009 growth trend among the 10 most frequent author keywords, followed by the rapid growth of “functional dyspepsia,” showing a distinct J-shaped curve, subsequently trailed by “anxiety” and “dyspepsia.” Conversely, “stress” and “gastric motility” exhibited a flatter trajectory. Trend topic analysis, a potent tool for mapping and visualizing the integration of trends within a particular field, amalgamated previous research streams (Dai et al., 2022). In addition, the keyword plus analysis provided an independent supplement revealing additional paper details on the relevant topic (Sun et al., 2012). Thus, a trend topic graph related to FD and anxiety/depression emerged based on keyword plus frequency (Figure 8D). Set at a minimum occurrence frequency of five times and three words per year. The analysis yielded the following findings: “psychological factors” had the longest duration (12 years, from 2007 to 2019), and keywords such as “health,” “non-ulcer dyspepsia,” “double-blind,” “hospital anxiety,” and” accommodation” continued to rage. The frequencies of “functional dyspepsia,” “depression,” and “anxiety” peaked in 2014, 2018, and 2019, respectively. Notably, “mast cells” surfaced in recent years, persisting as a significant topic in FD and anxiety/depression, culminating in a peak in 2021.

## 4. Discussion

### 4.1. Global contributions to the research areas associated with FD and anxiety/depression based on bibliometric analysis

A mounting body of evidence underscores the intricate link between FD and anxiety and depression (Wang et al., 2022; Carpinelli et al., 2023; Chen et al., 2023). As the precise etiology of FD remains elusive and treatment options are limited, a thorough scientific analysis of publications concerning FD, anxiety, and depression might provide new ideas for FD treatment and research. Therefore, this study employed a bibliometric approach suited for evaluating extensive scientific literature within a specific research domain (Aggarwal et al., 2016). This method explores global trends and research hotspots in publications concerning FD and anxiety/depression over the past two decades. Unlike meta-analysis, bibliometrics, with its more lenient screening criteria, obtains more data support, leading to more objective and comprehensive outcomes. Additionally, the cluster and timeline analysis functions offered by the CiteSpace bibliometric tool facilitate quick access to key information, elucidating development trends and hotspots. Furthermore, the graphic visualization inherent to bibliometrics provides a more intuitive interpretation of significant findings, superior to systematic reviews and meta-analyses. In this study, using CiteSpace, VOSviewer, Microsoft Excel, and an online analysis platform, 338 papers related to FD and anxiety/depression were retrieved and screened from the WOSSC, effectively capturing current research hotspots and emerging trends within this field. The findings revealed a fluctuating yet predominantly exponential growth trajectory in publications over the last two decades, indicative of escalating attention toward this research sphere, gradually becoming a research hotspot.

Asia, the US, Australia, and various European countries prominently emerge as the primary regions for publishing articles on FD and anxiety/depression. Among these, China leads with a significantly higher volume of publications, accompanied by a robust presence of research institutions and scholars in this subject area. While the US, Australia, and Germany exhibit relatively fewer publications, their high citations per article indicate a substantial impact within the field. Importantly, the development of this area varies significantly in terms of country/region and cooperation intensity. China is the sole developing country featured in the top 10 countries, while institutional collaboration centered on the Chengdu University of Traditional Chinese Medicine predominantly involved Chinese institutions. Newcastle University and Mayo Clinic predominantly collaborate with institutions from Australia and Sweden. Considering the epidemiological and clinical model differences between Eastern and Western countries within this field (Mahadeva and Ford, 2016), fostering enhanced exchanges and cooperation among these countries will contribute to advancements in this field.

Academic journals serve as important carriers of knowledge dissemination and shaping specific practice fields (Podolsky et al., 2012). The quality and reputation of a journal play a crucial role in disseminating research within a particular field. The IF is an index that reflects a journal’s influence, encompassing citation rate, academic reputation, and overall paper quality within the field (Xu X. et al., 2022). Of the 10 journals, namely *GASTROENTEROLOGY* and *CLINICAL GASTROENTEROLOGY AND HEPATOLOGY,* both from the USA, with the highest number of publications on FD and anxiety/depression, had an IF of ≥10. These journals, which represent a high level of research in this field, will receive more attention from researchers aiming to further understand FD’s relationship with anxiety/depression. The discipline of the citing journal in the dual-map overlay and the discipline of the cited journal at the other end of the citation link can provide inspiration for research in a specific field (Chen et al., 2014). Our findings indicate that disciplines pertinent to FD and anxiety/depression primarily focused on clinical medicine, nursing, and psychology. Consequently, fostering robust interdisciplinary connections stands as a means of bolstering and advancing research in this field.

### 4.2. Hotspot evolution and current trends in research fields associated with FD and anxiety/depression

The early references within this field focused on “#10 Burden” and “#11 Anxiety.” Numerous studies have substantiated the correlation between social psychological factors and FD across various countries. For instance, a US-based study compared dyspepsia symptom scores and mental status in 151 patients with FD and 90 healthy participants in a primary care clinic. The results revealed that patients with FD exhibited elevated symptom scores and increased mental burden compared with healthy participants (Jones et al., 2005). Fu et al. conducted a cross-sectional study on the prevalence of anxiety and depressive symptoms among patients with somatic disorders across 12 tertiary hospitals in different Chinese cities. The results indicated that among 305 patients with FD, 56% exhibited definite symptoms of depression and up to 70% had definite symptoms of anxiety (Fu et al., 2007). Furthermore, beyond the aforementioned countries, a study encompassing 853 patients with dyspepsia from primary and secondary medical institutions in Germany, Hungary, Italy, Poland, South Africa, and Spain revealed that apart from abdominal pain and dyspepsia, approximately 18–43% of individuals in each country experienced anxiety (Halling et al., 2008). During that period, the high prevalence of anxiety was thought to be potentially linked to abnormal antral food retention in FD or to lowered thresholds for gastric discomfort, pain, and compliance (Lorena et al., 2004; Van Oudenhove et al., 2007).

As research has progressed, the focal points in this field have shifted toward psychological comorbidity, cerebral activity, sleep disorders, and sex-related difference. The emergence of the term “psychological comorbidity” seems to better illustrate the close association between FD and anxiety and depression. This relationship has gained specificity since the introduction of the Rome III diagnostic criteria in 2006, which classified FD into PDS and EPS. A cross-sectional study from Switzerland in 2009, as representative literature, suggested that anxiety was associated with FD and PDS but not EPS (Aro et al., 2009). However, the question remains whether centrally mediated anxiety regulates the underlying mechanisms of PDS, encompassing fundic disaccommodation and visceral hypersensitivity. On the contrary, certain studies have indicated PDS’s association with depression (Hsu et al., 2009; Clauwaert et al., 2012), proposing that an abnormal brain-gut reciprocal neural connection could elucidate these symptoms’ fundamental pathophysiological mechanism (Hsu et al., 2009). Some studies have even indicated severe depression to be exclusive to EPS (Jamil et al., 2016). While these studies have deepened our understanding of the relationship between FD and anxiety/depression, their cross-sectional nature curtails causal explication. Consequently, researchers turned to cohort studies and basic experiments related to it. A Swedish longitudinal study on 887 individuals revealed that baseline anxiety, rather than depression, increased the likelihood of developing FD by 7.6 times over a decade (Aro et al., 2015). Additionally, an 11-year historical cohort analysis from Taiwan, China indicated a 70% higher incidence rate of FD in the depression group compared with the control group (Kao et al., 2021). These findings underscore anxiety and depression’s potential role in the pathogenesis of FD, partly explained by the brain-gut axis (BGA). The BGA facilitates bidirectional communication between the central nervous system (CNS) and the sensory and effector nerves of the gastrointestinal tract. This enables direct communication between the enteric nervous system (ENS) and the CNS through “bottom-up” (ENS to CNS) and “top-down” (CNS to ENS) pathways (Tait and Sayuk, 2021). Consequently, the BGA might modulate gastrointestinal function descendingly and elicit gastrointestinal responses to emotions. The hypothalamic-pituitary-adrenal (HPA) system, a BGA component, produces adaptive responses to stressors by regulating multiple hormones. Acute stress can increase intestinal epithelial permeability, a potential key to visceral hypersensitivity (Tait and Sayuk, 2021). Corticotropin-releasing factor (CRF) within the HPA system emerges as a pivotal mediator connecting stress with increased intestinal permeability (Camilleri et al., 2012). Moreover, Tominaga et al. proposed that up-regulated serotonin transporter levels in the midbrain and thalamus might result in the pathogenesis of FD through brain-gut interactions (Tominaga et al., 2015).

FD can also cause anxiety and depression. Studies have found that individuals who develop chronic dyspepsia symptoms in adolescence and young adulthood are at an increased risk of anxiety disorders (Rippel et al., 2012). A 1-year prospective study by Koloski et al. among Australians revealed that while one-third of individuals experienced anxiety and depression before functional gastrointestinal symptoms onset, two-thirds experienced gastrointestinal symptoms prior to anxiety and depression (Koloski et al., 2016). This suggests that gastrointestinal factors might play a more crucial role as initiating agents. In animal experiments, Liu et al.’s previous work demonstrated that transient stomach or colon inflammation or injury, even when resolved, could result in long-term hypersensitivity and motor abnormalities (Liu et al., 2008). Based on this, they induced FD in male Sprague–Dawley (SD) rats via neonatal gastric stimulation (primarily intragastric administration of 0.1% iodoacetamide [IA] over 6 days to 10-day-old rats) and examined anxiety and depressive behaviors at 8–10 weeks of age. They observed that compared with the control group, IA-treated male SD rats (FD rat model) displayed anxiety and depression-like behavioral changes, alongside heightened CRF expression in the hypothalamus and increased stress responsiveness of the HPA axis (Liu et al., 2011). Interestingly, antalarmin, a corticotropin-releasing factor type 1 (CRF1) receptor antagonist, ameliorated depressive-like behavior in IA-treated male rats, suggesting CRF1 receptor mediation in the male FD rat model’s depressive-like behavior. Studies by Luo et al. yielded similar outcomes, showing evident anxiety- and depression-like behaviors and a hyperactive HPA axis specifically in female but not male FD rat models post IA treatment, thereby proposing a sex-specific gastrointestinal inflammation-induced neuroendocrine (HPA axis) pathway for anxiety/depressive behavioral shifts (Luo et al., 2013). Furthermore, Cordner et al. ascertained that a similar FD rat model exhibited increased vagal activity representing the gastric distention responses. Interestingly, vagotomy (bilaterally subdiaphragmatic) not only attenuated the pain response to gastric distention but also reversed the depression- and anxiety-like phenotype and reduced corticotrophin-releasing hormone (CRH) expression in the central amygdala (Cordner et al., 2021). This suggests that vagal activity could augment pain and anxiety- and depression-like behaviors by inducing changes in CRH signaling in the amygdala. in summation, FD might contribute to anxiety and depression onset by inner secretory activation (HPA axis) and heightened vagus nerve activity (associated with gastric distention response).

Psychiatric disorders have highlighted the significance of altered functional neuronal activity within the brain (Zhang and Raichle, 2010), with a growing emphasis on resting-state brain activity abnormalities. For example, Van Oudenhove et al., using brain H_2_^15^O-positron emission tomography, discovered that anxiety in patients with FD was negatively correlated with resting brain activity in pregenual anterior cingulate (pACC) and midcingulate regions, while positively correlated with dorsal pons activity (Van Oudenhove et al., 2010). Researchers designed a macaque version of an approach-avoidance decision-making task assessing anxiety and depression in humans to understand the circuitry-level mechanism of pACC-related anxiety. Employing multi-electrode recording and cortical microstimulation, researchers explored pACC function in macaques during task performance (Amemori and Graybiel, 2012). They identified pACC neurons representing positive motivation (P-type units) and negative subjective value (N-type units), with similar distributions in most pACC regions, except for a higher proportion of N-type units in the ventral bank of the cingulate sulcus. Microstimulation in this region enhanced negative decision-making, reversible by anti-anxiety drugs, affirming pACC’s anxiety regulatory role. Cingulate cortex activity’s link with anxiety is influenced by the degree of threat, with anxiety positively associated with pACC activity during moderate threat periods and negatively during high threat periods (Straube et al., 2009). This hints at visceral stimulation studies simulating high-threat environments for individuals with FD. Patients with FD, particularly refractory cases, are also often prone to sleep disturbances, in addition to psychological distress (Yu et al., 2013; Jiang et al., 2015; Li et al., 2018). These factors interrelate, as evidenced by a Japanese clinical study showing significant associations between sleep disturbances dyspepsia, and depressive symptoms in patients with FD compared with healthy controls (Futagami et al., 2013). Anxiety has emerged as an independent risk factor for sleep disorders (Li et al., 2018; Park et al., 2021), with sleep disorders even increasing FD risk by 3.3 times (Su et al., 2021). Sex-related differences are observed in the relationship between FD and anxiety and depression. Women with FD generally exhibit higher anxiety and depression scores compared to men with FD (Choi et al., 2017; Huang Z. P. et al., 2022), possibly due to more pronounced cognitive-affective processing dysfunction in female patients with FD (Welen et al., 2008).

In the past three years, the timeline view of co-cited references and keyword trend analysis indicates an emerging research hotspot within the field, that is, duodenal low-grade inflammation, particularly duodenal eosinophilia and mast cell involvement, which is pivotal in the pathogenesis of FD (Wauters et al., 2020). Majorly, mucosal eosinophils, a major inflammatory subtype of FD (Wauters et al., 2020), originate from pluripotent bone marrow stem cells and migrate to the gastrointestinal tract via peripheral blood (Powell et al., 2010). Duodenal eosinophilia in patients with non-ulcer dyspepsia was first reported by Talley et al. (2007). Subsequently, numerous studies have reported similar findings in patients with FD (Walker et al., 2009, 2010; Nwokediuko et al., 2013; Walker et al., 2014; Ronkainen et al., 2019), highlighting its non-accidental phenomenon. Meanwhile, duodenal eosinophilia is more prevalent in patients with PDS (Walker et al., 2010, 2014). Postprandial fullness is associated with delayed gastric emptying (solid and liquid phase) (Sarnelli et al., 2003), which is linked to eosinophilic gastroenteritis (Caglar et al., 2012), offering insights into subtype-related duodenal eosinophilia. Increased degranulation of duodenal eosinophils in some patients with FD (Du et al., 2016; Vanheel et al., 2018) suggests activation, potentially impairing epithelial barrier function. Mechanisms include major basic protein-induced occludin (a tight junction molecule) down-regulation (Furuta et al., 2005), CRH release, mast cell activation, and barrier dysfunction (Zheng et al., 2009; Wallon et al., 2011). Mast cell proliferation and activation in the intestinal tract of patients with irritable bowel syndrome who were predominantly diarrheal have been validated (Guilarte et al., 2007; Lee et al., 2008). Similarly, Vanheel et al. reported mast cell infiltration in the duodenal mucosa of patients with FD (Vanheel et al., 2014), potentially related to impaired intestinal barrier function. This is because mast cells can be activated by soluble mediators such as stem cell factors and major basic proteins derived from duodenal eosinophils, thereby promoting the release of pancreatic proteases, histamine, and prostaglandin D2 (Powell et al., 2010). Eosinophil and mast cell infiltration might affect the ENS. For example, Cirillo et al. found significant eosinophil and mast cell infiltration in the duodenal submucosa, and the number of these cells was negatively correlated with calcium transient amplitudes measured in the submucosal ganglia, confirming the contribution of these two types of inflammatory cell infiltration to submucosal plexus neuron damage (Cirillo et al., 2015). Additionally, the emotional state of patients with FD might be directly affected by low-grade gastrointestinal inflammation. Yuan et al. linked duodenal mucosa mast cell count and degranulation to The Hospital Anxiety Depression Scale (HADS)-A(anxiety) and HDS-D(depression) scores in patients with PDS and EPS (Yuan et al., 2015). A 10-year follow-up study in Switzerland by Ronkainen et al. revealed that study population (regardless of FD status) with duodenal eosinophilia [more than 23 eosinophils in the bulb (D1)] at baseline had a nearly five-fold increase in clinical anxiety after follow-up, suggesting that duodenal intestinal eosinophilia might be an anxiety-related mechanism independent of FD (Ronkainen et al., 2021). Indeed, psychiatric comorbidities are prevalent in patients with non-esophageal eosinophilic gastrointestinal disease (Reed et al., 2022). A possible explanation is that patients with gastroenteropathy characterized by eosinophilic infiltration are prone to greater stigma, which in turn induces more anxiety and depression (Guadagnoli et al., 2017; Guadagnoli and Taft, 2020). Despite encouraging results, they will only be more convincing if the anxiety is relieved by the alleviation of low-grade duodenal inflammation. Limited clinical trial data exist; however, an animal study revealed that the mast cell stabilizer ketotifen significantly alleviated pain and mood disturbances in FD rat models with anxiety-like or depression-like manifestations and with an increase in mast cell infiltration (Cordner et al., 2021). In summary, the current study suggests that low-grade duodenal inflammation (especially increased eosinophil or mast cell infiltration in the duodenum) significantly affects FD pathogenesis and anxiety/depression. Further research is warranted to confirm this association and promote precise FD treatment.

### 4.3. Current applications and prospects for the future

Researchers have actively pursued novel treatments for FD based on the understanding of its relationship with anxiety/depression, yielding promising initial outcomes. Antidepressants and anti-anxiety drugs have emerged as treatment options, particularly for patients with psychiatric comorbidities. Imipramine, a tricyclic antidepressant, alleviated refractory FD symptoms (Cheong et al., 2018). Mirtazapine, a tetracyclic antidepressant, significantly improved early satiety and anxiety in adults and adolescents with FD (Tack et al., 2016; Iglesias-Escabi et al., 2022). The compound preparation of flupentixol and melitracen simultaneously improved gastrointestinal symptoms, anxiety, and depression (Huang Q. et al., 2022). Psychotherapies, such as cognitive-behavioral therapy and self-sleep interventions, demonstrated therapeutic potential akin to psychotropic drugs (Orive et al., 2015; Tavakoli et al., 2020; Kinsinger et al., 2022). Two-week percutaneous ear vagus nerve stimulation, targeting the vital gut-brain communication conducted by Zhu et al. effectively controlled dyspeptic symptoms and mood disturbances (Zhu Y. et al., 2021), validated by recent animal experiments (Hou et al., 2023). Japanese herbal medicine Yokukansan attenuated gastric hypersensitivity in FD rats by attenuating eosinophil-associated micro-inflammation (Duan et al., 2022). Chinese herbal medicine and acupuncture therapies also yielded positive outcomes in patients with FD with anxiety/depression through distinct mechanisms (Zhao and Gan, 2005; Luo et al., 2015; Su et al., 2018; Chen F. et al., 2022). However, treatment efficacy, particularly for drug therapies, is constrained by limited sample sizes and the lack of prospective studies. Therefore, conducting more prospective studies on FD and anxiety/depression is valuable to clarify the causal relationship between FD and anxiety/depression and can guide treatment focus. While antidepressants show therapeutic potential, patient stigma often undermines adherence (Yan et al., 2021). Future drug development should prioritize higher acceptance or explore alternatives such as neuromodulation methods (e.g., transcutaneous auricular vagus nerve stimulation), preferred for their non-invasive nature and easy implementation. The discovery of duodenal low-grade inflammation offers fresh insights into FD and mood disorders, facilitating novel treatment avenues (e.g., mast cell stabilizers or potential eosinophil stabilizers). Further research on low-grade duodenal inflammation could transform symptom-based empirical treatments in patients with FD with comorbid mood disorders into precise therapies targeting underlying pathological changes in the stomach and duodenum.

### 4.4. Strengths and weaknesses

While previous research has conducted comprehensive bibliometric analysis on FD, some possibly might overlook the importance of individualized treatment for FD, failing to emphasize the inappropriateness of antidepressants for patients with FD without mood disorders. Furthermore, prior bibliometric methods used were relatively limited in scope and lacked validation. This study stands out by identifying a novel trend in FD research related to anxiety/depression through bibliometric methods, offering fresh insights for patients with these comorbidities (e.g., the potential link between duodenal inflammation and anxiety/depression). Employing multiple bibliometric analysis tools enhances the accuracy and reliability of our identified research hotspots. However, the study has certain limitations. First, the exclusion of publications before 2003 due to their scarcity might miss critical earlier studies. Second, automated database updates might introduce discrepancies in results. Finally, current software limitations hinder cross-database analysis, confining this study to WOSCC for literature screening, and possibly omitting relevant literature. Despite these constraints, the current bibliometric analysis effectively guides scholars toward research hotspots related to FD and anxiety/depression and emerging trends within the field.

## 5. Conclusion

The bibliometric analysis underscores the promising trajectory of research on FD and anxiety/depression, displaying exponential growth, particularly in the past 2 years. The analysis identifies the major countries/regions, institutions, authors, journals, co-cited references, and keywords within this research domain. It was observed that studies associated with FD and anxiety/depression focused on psychological comorbidity, cerebral activity, sleep disorder, and sex-related difference as key focal points in FD-anxiety/depression investigations. In recent years, it has been found that low-grade duodenal inflammation might play a crucial role in FD pathogenesis or anxiety/depression. Clinical trials and basic experiments focusing on the relationship between increased eosinophil or mast cell infiltration in the duodenum and FD, as well as anxiety/depression, might become a prominent direction of focus in this field.

## Data availability statement

The original contributions presented in the study are included in the article/supplementary material, further inquiries can be directed to the corresponding author.

## Author contributions

QH and HJ defined the research plan. QH finished the first draft. HY and QL collected and sorted the data. SG, YZ, and ML performed bibliometric analysis and drew diagrams. YL and HJ made important revisions to the paper. All the authors performed their tasks effectively and consented to manuscript submission.
